# Oxidation and fragmentation of plastics in a changing environment; from UV-radiation to biological degradation☆

**DOI:** 10.1016/j.scitotenv.2022.158022

**Published:** 2022-08-12

**Authors:** A.L. Andrady, P.W. Barnes, J.F. Bornman, T. Gouin, S. Madronich, C.C. White, R.G. Zepp, M.A.K. Jansen

**Affiliations:** a Chemical and Biomolecular Engineering, North Carolina State University, Raleigh, NC, USA; b Biological Sciences and Environmental Program, Loyola University New Orleans, New Orleans, LA, USA; c Food Futures Institute, Murdoch University, Perth, Australia; d TG Environmental Research, Sharnbrook, Bedfordshire, UK; e Atmospheric Chemistry Observations and Modeling Laboratory, National Center for Atmospheric Research, Boulder, CO, USA; f Exponent, Inc, Bowie, MD 20715, USA; g ORD/CEMM, US Environmental Protection Agency, Athens, GA, USA; h School of BEES, Environmental Research Institute, University College Cork, Cork, Ireland

**Keywords:** Plastic, UV radiation, Photo-oxidation, Fragmentation, Environmental persistence

## Abstract

Understanding the fate of plastics in the environment is of critical importance for the quantitative assessment of the biological impacts of plastic waste. Specially, there is a need to analyze in more detail the reputed longevity of plastics in the context of plastic degradation through oxidation and fragmentation reactions. Photo-oxidation of plastic debris by solar UV radiation (UVR) makes material prone to subsequent fragmentation. The fragments generated following oxidation and subsequent exposure to mechanical stresses include secondary micro- or nanoparticles, an emerging class of pollutants. The paper discusses the UV-driven photo-oxidation process, identifying relevant knowledge gaps and uncertainties. Serious gaps in knowledge exist concerning the wavelength sensitivity and the dose-response of the photo-fragmentation process. Given the heterogeneity of natural UV irradiance varying from no exposure in sediments to full UV exposure of floating, beach litter or air-borne plastics, it is argued that the rates of UV-driven degradation/fragmentation will also vary dramatically between different locations and environmental niches. Biological phenomena such as biofouling will further modulate the exposure of plastics to UV radiation, while potentially also contributing to degradation and/or fragmentation of plastics independent of solar UVR. Reductions in solar UVR in many regions, consequent to the implementation of the Montreal Protocol and its Amendments for protecting stratospheric ozone, will have consequences for global UV-driven plastic degradation in a heterogeneous manner across different geographic and environmental zones. The interacting effects of global warming, stratospheric ozone and UV radiation are projected to increase UV irradiance at the surface in localized areas, mainly because of decreased cloud cover. Given the complexity and uncertainty of future environmental conditions, this currently precludes reliable quantitative predictions of plastic persistence on a global scale.

## Graphical Abstract

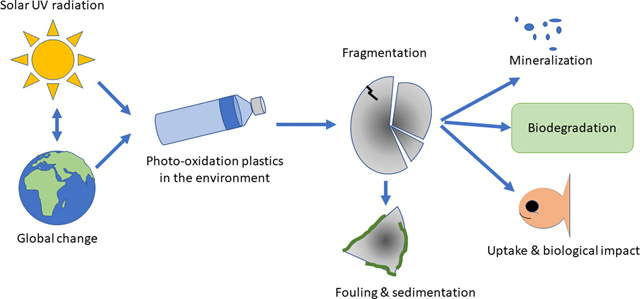

## 1. Introduction

Recent research has highlighted the global ubiquity of plastic debris, including microplastic (MP) particles, across the planet and particularly in the ocean. The presence of plastic materials in the environment represents a particular concern given their inherent recalcitrance. Their environmental persistence, together with potential fragmentation into Micro- and possibly nanoplastic (NP) particles, may pose both short- and long-term risks to the environment and human health (Koelmans et al., 2019; Borrelle et al., 2020; Stubbins et al., 2021; Mehinto et al., 2022). Comprehensive assessments of the risks associated with plastic pollution are needed to address a variety of concerns including: the direct physical effects related to encounters between organisms and plastic debris in natural ecosystems, indirect effects, such as the potential alterations that plastic particles may cause to soil and sediment systems, the potential role of plastic debris as vectors of transport for pathogens and invasive species, and the risks associated with chemicals that may leach from plastic debris. Given the wide variety of plastic products used in commerce and in other applications, it is important to recognize that in addition to the base polymers, plastics typically contain catalyst residues, unreacted monomers, as well as intentionally added chemicals including plasticizers, dyes, antioxidants, flame retardants and/or UV stabilizers at varying concentration levels (Sendra et al., 2021; Gouin, 2021; Fauser et al., 2022). Given their high equilibrium distribution coefficients in plastic/seawater system plastic debris in the ocean pick up and concentrate, hydrophobic pollutants dissolved in seawater (Andrady, 2011). Yet, the environmental consequences of this process are likely to be minimal given that abundant organic debris would similarly partition such pollutants. Due to their intrinsic heterogeneity with respect to particle shape, size and composition, together with uncertainties concerning environmental exposure doses, potential adverse biological effects are not well established, and it is currently not possible to conduct a robust risk assessment of waste plastics (Coffin et al., 2021).

Prominent among the various challenges towards an improved understanding of the environmental fate of MPs, is the incomplete knowledge of degradation and fragmentation of plastic waste, especially in aqueous environments (Ward and Reddy, 2020). This knowledge gap is not well addressed, and hampers environmental impact studies. Degradation constitutes any chemical change that compromises the desirable properties of the plastic material. Photo-oxidation by solar UVR is generally regarded the most significant mechanism of environmental degradation (Masry et al., 2021). Biodegradation of plastics by microorganisms has also been reported (Shah et al., 2008; Anjana et al., 2020). Relative to photo-oxidation, biodegradation is much slower and is not thought to remove plastics from the environment in a readily observable timescale. Solar UV radiation is thus the primary factor that drives generation of MP fragments.

The Montreal protocol on substances that deplete the ozone layer, and its scientific assessments by the Environmental Effects Assessment Panel of UNEP, have direct relevance for managing plastic pollution. From one perspective, the implementation of the Montreal Protocol has resulted in a global reduction in the photo-oxidation and subsequent generation of MPs and NPs from plastic debris. Large increases in the amount of solar UVR reaching the earth’s surface (due to further deterioration of the stratospheric ozone layer) were avoided by the implementation of the Montreal Protocol (the World avoided; McKenzie et al., 2019). The Protocol may also have resulted in reduced efficacy of any potential UV-driven photo-mineralization process, potentially extending the environmental persistence of MP and NP generated from plastic debris. However, biomineralization is by far the predominant mechanism of mineralization of plastics in the environment. Interactive interactions between solar radiation and climate change (Bais et al., 2019; Neale et al., 2021) will further impact the oxidative fragmentation rates of plastics, particularly in areas of high UV radiation due to decreased cloud cover or high elevation. Conversely, plastics may also indirectly contribute to global warming, if only by being a significant component of the global carbon budget (Dees et al., 2020). Thus, there is a network of complex, and potentially counterintuitive, interactions between UV-radiation, climate change and plastics, influencing their environmental fate, transport and exposure. The aim of this paper is to identify the role of solar UVR, the dominant driver of photo-oxidation, in determining formation and persistence of MP within the context of a rapidly changing climate.

## 2. Dispersal of plastic in the environment

Central to determining the environmental fate, impacts and global distribution of post-consumer plastic debris, is the need to better characterize and quantify their environmental persistence, taking into account all different modes of potential degradation and their mechanisms. Plastic litter may range from macroplastic debris to MP and NP (Zhu, 2021). The evaluation of their dispersal and distribution also represents an important area of research that informs persistence studies (Stubbins et al., 2021; Gouin, 2021), since both the propensity towards poor disposal (or littering) and the UV radiation environments are location-dependent. Given the extensive use of polyethylene [PE], polypropylene [PP] and poly(ethylene terephthalate) [PET] in packaging, as well as the use of PET and polyamide (nylon) fibers in textiles and in fishing equipment, it is not surprising that these polymers represent the most commonly encountered types of plastic litter. These polymers are reported as urban litter, air-borne particles, floating debris and beach debris, in both marine and freshwater systems. Microfibers (MFs) are especially abundant in the environment, although it needs to be recognized that a very large majority (~80 %) of these fibers are cellulosic (Suaria et al., 2020). These pollutants are mostly derived from synthetic textiles that shed microfibers during use (De Falco et al., 2020) and after disposal (Sun et al., 2021). Synthetic textile fibers are made of, among others, PET and Nylon, both denser than sea water, and likely to be most abundant in the sediment. While limited sampling of sediments has been reported (Gago et al., 2018) there are currently no global estimates of the abundance of MFs in sediment.

Abundance data coupled with model estimates suggest that the global mass of floating plastic debris in the ocean represents only a very small percentage of their estimated annual influx based on resin production volumes (Lebreton et al., 2019; Dees et al., 2020; Stubbins et al., 2021), triggering the question of ‘where is the missing plastic?’ The fraction perceived as ‘missing’, however, may represent an artifact of how the samples have been collected, whereby the majority of studies limit their analysis to floating plastic in the ocean, collected from surface water using a sampling protocol that is constrained by the mesh size of the collecting gear employed. Thus, these approaches do not fully appreciate plastics in terrestrial, freshwater and atmospheric environments, nor capture smaller MPs or potential NPs. Furthermore, while common litter of PE and PP float in water, biofouling and hetero-aggregation of debris (Van Melkebeke et al., 2020; Wu et al., 2020) invariably results in their sinking into the deep-water column, precluding their sampling using methods that focus only on surface waters. Biofouling involves the colonization of plastic surfaces by a biofilm of microorganisms, algae, and small shelled species (Amaral-Zettler et al., 2015; Roager and Sonnenschein, 2019). Hetero-aggregation refers to adhesion of particles to each other or to biomass via bacterial exopolymer secretions, resulting in the formation of larger aggregates of MP with organic and inorganic detritus (Leiser et al., 2021). Both processes tend to decrease the buoyancy of plastics, causing sedimentation (Besseling et al., 2017; Wu et al., 2020; Kaiser et al., 2017). Some small fraction of the missing plastics, however, might also be understood in terms of geochemical cycles, taking into account both photo-and (bio)degradative processes that results in mineralization.

Although it has been suggested that approximately 80 % of ocean plastic has a land-based origin (Lebreton et al., 2019), the potential scope of estuarine and riverine transport into the open ocean (Tramoy et al., 2020; Lebreton and Andrady, 2019), and mechanisms that return plastic from the ocean to coastal beaches (Onink et al., 2021), are not well understood. Schernewski et al. (2021) estimated the aqueous-phase residence time of 20–500 μm PE and PP particles discharged from wastewater treatment systems into the watershed of the Baltic Sea region to be only about 14 days before they either sink or are washed ashore on to local beaches. Recently, it was suggested that the residence times of floating plastic (and exposed to UVR) are years rather than days as previously thought (Weiss et al., 2021). Thus, coastal environments and deep-ocean sediments likely represent important sinks for MPs (Woodall et al., 2014; Harris, 2020), critically informing studies on the environmental fate and transport of plastics. Both the interchange of plastic between coastal water and beaches and the sinking of plastics debris in the water column, are processes that significantly affect their exposure to solar UVR and high temperature (Andrady, 2022). It can therefore be assumed that these processes will directly influence the rates of oxidation and the subsequent fragmentation of plastic.

## 3. Photo-oxidative action of UV radiation on plastics debris

Secondary MPs are derived from degradative fragmentation of plastic debris, mostly as a result of their weathering on exposure to solar UV radiation (Andrady, 2017; Vega et al., 2021). Photo-oxidation weakens and embrittles the plastic, and fragmentation occurs when the plastic is subjected to mechanical stresses in the environment. In the ocean environment, wave action, swelling-deswelling, abrasion with sand, and encounters with marine organisms serve to fragment weakened plastics.

**Fig. 1.**
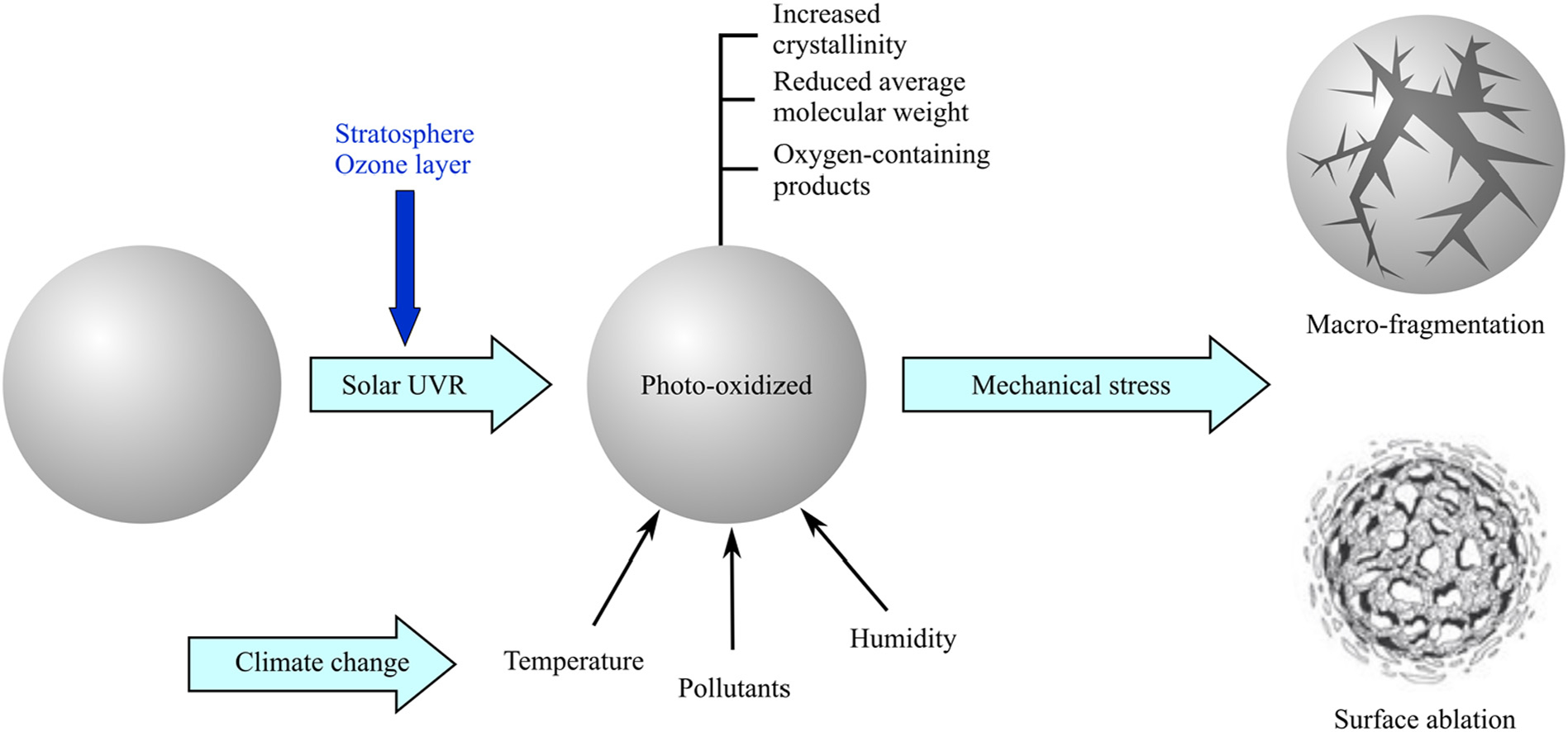
Schematic overview of photo-oxidative reactions, starting with a virgin plastic sphere (left), and leading to fragmentation (top right) and/or surface ablation (bottom right) of plastics.

The chemistry of photo-oxidation of common plastics has been well-studied (Rodriguez et al., 2020; Grause et al., 2020) with most research focused on early oxidation of the material during its useful service life (i.e., of interest for the plastics manufacturing industry, as well as for regulatory bodies assessing performance standards). The free-radical reaction sequence involved in photo-oxidation is similar to that for the auto-oxidation of common olefins and unsaturated hydrocarbons, except for the restricted mobility of macromolecular radical species in the solid-phase (Garton et al., 1980). With thick plastic samples, the rate of reaction often tends to be diffusion-controlled, as a result of oxidation occurring largely in a 500-to-900-micron thick surface layer of the sample (Fig. 1), depending on the type of polymer (Shyichuk et al., 2004; Nagai et al., 2005; Andrady et al., 2022). Initial products of oxidation reactions are polymeric hydroperoxides that undergo photo- or thermolysis to create macroradicals that can react with oxygen dissolved in the matrix; hence the autocatalytic nature of photo-oxidation in these plastics. Photo-oxidation of PE and PP mainly yields carbonyl compounds, chain unsaturation and carboxylic acid products, where some of these are chromophoric, and therefore initiate further photo-initiation. Typically, weathering increases the absorption coefficients for UVR (e.g., via formation of carbonyl chromophores, or surface-induced bathochromic shifts). Therefore, it is possible that UVR-mediated oxidation makes plastics even more susceptible to subsequent solar radiation (Hakkarainen and Albertsson, 2004). While this putative, positive feedback cycle may result in rapid and full fragmentation, it remains largely unclear if it also leads to complete mineralization (Ward et al., 2019; Zhu et al., 2020).

Closely associated with photo-oxidation of polyolefins are crosslinking and chain-scission reactions, of which the latter predominates (Gewert et al., 2015; Pickett, 2018). Scission reactions occur both due to reactions mediated by oxy-radicals formed when polymer hydroperoxides decompose, as well as via Norrish photo-reactions of carbonyl species formed as products of oxidation. The average molecular weight of the plastic therefore decreases upon oxidation, and so does the mechanical integrity of the material. Oxidation of semi-crystalline PE and PP, the dominant plastic types in the environment, occurs almost exclusively in the amorphous fraction of the polymer (Bracco et al., 2018; Rodriguez et al., 2020). Resulting surface pits or cracks (Ter Halle et al., 2017; Cai et al., 2018), may upon further exposure to UVR, propagate into the bulk of the material, fragmenting the plastic into several daughter fragments (Fig. 1). Photo-oxidation merely weakens the polymer material, but the generation of smaller fragments or separate MPs typically requires additional mechanical stresses (Karlsson et al., 2018; Song et al., 2017). In the ocean or coastal environments, wave action is a significant source of mechanical stress, while on land either wind or interactions with animals, may serve this function. Only a minimal force is likely to be needed to fragment highly photo-oxidized, embrittled plastic as mere handling of embrittled plastics results in fragmentation (Corcoran et al., 2009; Song etal., 2017). Fragmentation of virgin, or mildly oxidized plastics, requires larger forces, but could potentially occur in the slush zone on beaches. Mechanical fragmentation of un-weathered, virgin plastic debris in the marine environment may occur through abrasion in the slush zone of the ocean with rocks and sand, and this process has been simulated in the laboratory (Chubarenko et al., 2020). Fragmentation of relatively unoxidized plastics is demonstrated by the formation of microfiber fragments during laundering of fabrics (Vassilenko et al., 2021) as well as by the crumbling of virgin plastics by ingesting crustaceans (Mateos-Cárdenas et al., 2020; Hodgson et al., 2018; Dawson et al., 2018), although the scale of the environmental relevance of the biological fragmentation process is unknown.

This sequence of photo-oxidation, weakening, embrittlement and fragmentation by mechanical stresses has been observed on land, in marine dry sediments (beach) (Corcoran et al., 2009) and in a few instances in surface water environments (Garvey et al., 2020; Alimi et al., 2022). Fragmentation results in increased specific surface area of the plastic sample, increasing the surface available for further photo-oxidation as well as biodegradation. Photo-degradation can continue, and at least in theory, yield significant amounts of MP and even NP, provided UVR is available to initiate the process.

As opposed to the macro-fragmentation process described above, a second, concurrent micro-fragmentation process occurs during weathering (Fig. 1). With diffusion-controlled oxidation that occurs in moulded or extruded plastic products, a highly degraded surface layer several hundred microns thick can be obtained in laboratory accelerated weathering (Andrady et al., 2022; Nagai et al., 2005; Ito et al., 2015). A similar layer is apparent in field collected plastics as well (Andrady et al., 2022). The oxidized surface has different mechanical properties from the bulk material and can readily delaminate or abrade away under mechanical stress to produce MPs as well as NPs by surface ablation. Extensively weathered plastic debris, collected from beaches and subjected to mild mechanical stresses in the laboratory yield microscale particles in large numbers, providing experimental support for surface layer ablation (Lambert and Wagner, 2016a, 2016b; Svedin, 2020). Laboratory-based accelerated weathering experiments, with plastics exposed to UVR and subsequently subjected to agitation with sand, similarly yield micro-fragments through surface ablation (Song et al., 2017). These accelerated weathering exposures can yield large numbers of micro- or nano-scale daughter fragments (~10^5^ to 10^6^ particles) per sq. cm of surface area of the plastic (Lambert and Wagner, 2016a, 2016b; Svedin, 2020). Consistent with the notion of degradation by surface-layer ablation, these daughter fragments have a size range of 10 s or 100 s of microns.

## 4. Photo-degradation and fragmentation of plastics in the ocean

Little is known about how photo- and thermo-oxidative reactions, and subsequent fragmentation, occur in natural environments, especially in air versus the aqueous environments. Three key differences between weathering in air versus in seawater have been identified (Andrady, 2011): a) lower temperatures of samples in water relative to those in air, slows down the rates of oxidation in seawater, as expected on the basis of the Arrhenius relationship; b) lower concentrations of dissolved oxygen in seawater, relative to in air, will slow oxidation rates (Andrady et al., 2022); c) sinking of plastics in the marine environment will result in the removal of the material from the photic zone, slowing down photo-initiation. Sinking into deep water or the sediment is often a result of a foulant layer developing on the surface of plastics that increases the apparent density of debris, making them less buoyant in seawater (Póvoa et al., 2021; Pabortsava and Lampitt, 2020). The biofilm may shield the plastic from solar UVR, further reducing photo-oxidative reactions (de Carvalho, 2017). The combination of these three factors, and especially the limited availability of oxygen, results in a marked retardation of photo-oxidation, and therefore of photo-fragmentation, of plastics in seawater. Common plastics weathered in seawater show only weak spectroscopic signatures of photo-oxidation (in FTIR studies), and tensile property measurements show a minimal decrease in mechanical properties compared to samples weathered in air (Arias-Villamizar and Vázquez-Morillas, 2018; Ojeda et al., 2011; Kalogerakis et al., 2017.) Consistent with this observation, no significant macro- or micro-fragmentation has been reported for plastics weathered in seawater under natural conditions, except in the case of thin film samples (Biber et al., 2019).

**Fig. 2.**
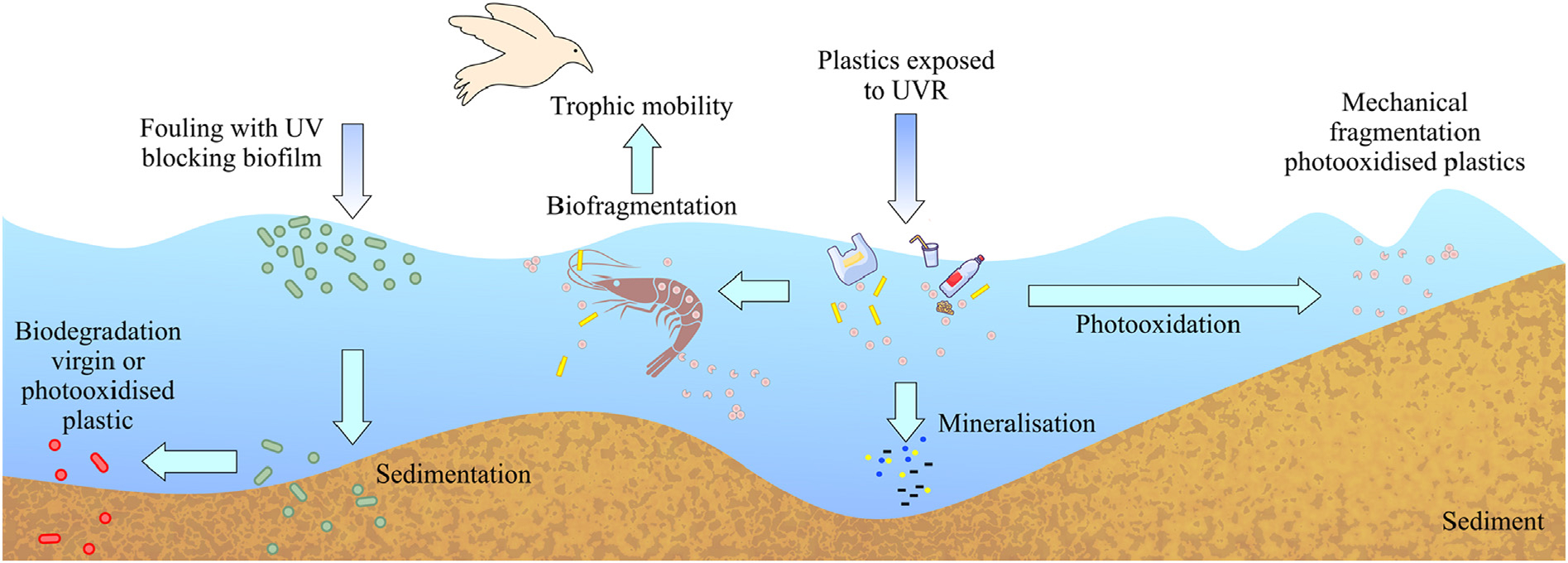
A schematic of the fate of plastic debris in the ocean environment. Exposure of plastics to UVR results in photooxidation and subsequently mechanical or biofragmentation. Fouling may decrease exposure to UVR through formation of a UV-blocking film, and through accelerated sedimentation.

Photo-assisted fragmentation is, however, likely to occur in seawater with those plastics already pre-weathered in air, land or beach (Fig. 2). To date, environmental sampling in surface waters has not resulted in the reporting of NPs or significant fractions of very small MPs. Although this may relate to the limitations of current sampling technologies, other possible explanations for the absence of these smaller plastics can be the sinking as well as the solubilization of particles. The latter suggestion is based on laboratory studies of plastics exposed to accelerated UV irradiation (Zhu et al., 2020; Ward et al., 2019), and implies that 100 % of EPS, PP and PE microplastics could be photo-chemically converted to Dissolved Organic Carbon (DOC) within 0.3, 0.3 and 0.5 years, respectively. Even ignoring that these DOC measurements may have included any NPs formed, the high amounts of UVR used in the studies do not allow a ready extrapolation of the data to the natural environment.

## 5. Photo-assisted fragmentation and a changing climate

Given the potential for significant changes in terrestrial solar UVR with ongoing and potential future changes in the stratospheric ozone layer and in the global climate, (Bais et al., 2019; Barnes et al., 2022), it is relevant to ask how these changes will impact the photo-assisted fragmentation of plastics in the environment. Both stratospheric ozone depletion and climate change can alter the solar UV-B (280–315 nm) and UV-A (315–400 nm) irradiance. In general, ozone depletion shifts the solar UVR spectrum to shorter wavelengths primarily in the UV-B. However, climate change may alter solar irradiance reaching the Earth’s surface without significantly affecting the spectral irradiance distribution (Barnes et al., 2022). Any increase in ambient air and seawater temperature under future climate scenarios will also accelerate the rate of photo-oxidation leading to fragmentation. Based on the Arrhenius relationship, an increase in oxidation of PE and PP can be modeled (Tamblyn and Newland, 1965; Therias et al., 2021), but a comparable model is not available for the fragmentation of these polymeric plastics. Assessing the impact of global changes on the exposure of plastics to UVR therefore requires an evaluation of the wavelength sensitivity of the UV-assisted fragmentation.

Spectral sensitivity data for plastics are available but limited to radiation-dependent oxidative changes such as discoloration, changes in the average molecular weight and mechanical integrity of weathered plastics (Andrady, 1997; Girois et al., 1996). These generally show that higher-energy regions of the solar spectrum, especially the UV-B component, are more efficient in causing photo-degradation. While these studies address wavelength-dependent changes in bulk mechanical properties of polyolefins, none directly pertain to their fragmentation in air or in seawater. There is no reason to expect an obvious correlation between spectral sensitivities for changes in bulk mechanical or physical properties and the photo-assisted fragmentation of polymers that also includes a mechanical stress. Nor are dose-response relationships, reciprocity information on intensity-dependence, or temperature coefficients established for the photo-assisted fragmentation of even the common plastics. Reported wavelength sensitivity data relate to virgin polymers, while post-consumer plastic debris typically includes a host of chemical additives, including UV stabilizers intended to modify photo-degradation processes. Non-availability of these critical data on fragmentation of plastic compounds that are widely used, precludes estimating the impact that ground-level UVR or increased temperatures may have on the rate of generation of MPs. These are very significant gaps in knowledge pertaining to photo-assisted fragmentation of plastics in the natural environment.

Both photo-assisted and purely mechanical generation of MPs contribute to the abundance of secondary MPs in the environment. However, there are no good criteria to identify the fractional contribution of each of these to the pool of MPs quantified in abundance studies. Nevertheless, qualitatively, large increases in the amount of UVR avoided by the implementation of the Montreal Protocol (the World avoided; McKenzie et al., 2019), would have increased rates of both photo-degradation and the photo-assisted fragmentation of plastics debris. With the Protocol ensuring no large increase in average solar UVR reaching the Earth’s surface due to human-related activity, any further acceleration of photo-fragmentation of plastics is unlikely in the future.

## 6. UV-stabilization and fragmentation

Typically, plastic products intended for outdoor use are compounded with an opacifier, such as rutile titanium dioxide, popularly used in rigid PVC, or with low levels of UV-stabilizers, mostly UV absorbers or free-radical quenchers (Rani et al., 2017; Petukhova and Fedorov, 2019; Wypych, 2020), to retard their oxidation. The efficacy of UV stabilizers has been well demonstrated in mitigating the oxidative effects of degradation of common plastics by UVR, ensuring full functionality of plastic products during their service life outdoors. It is reasonable to expect stabilizers to also mitigate both fragmentation and mineralization of plastic debris, even though there is no experimental evidence to support this. Thus, while the use of UV-stabilizers may improve durability of plastic products, a potential negative attribute is the persistence of plastic debris.

Omission of UV-stabilizers in plastic compounds will likely decrease product lifetime and increase photo-assisted fragmentation with a mixed environmental impact (Song et al., 2017). On the one hand, it may reduce amounts of larger, visible plastic debris in the environment. On the other hand, the omission may encourage faster fragmentation, leading to higher amounts of MPs and NPs in the environment (Ouyang et al., 2021). The strategy is not unlike the use of pro-oxidant additives in plastics to encourage oxidation and fragmentation in the environment. Technologies that catalyze photo-oxidation, using either modified polymers or transition metal compounds, are available and are known to accelerate photo-assisted fragmentation rates (Nabi et al., 2021). For instance, in the USA, degradable six-pack, can packaging is mandated. In these plastics the PE resin is substituted by a co-polymer of ethylene and 1 % of carbon monoxide, resulting in a weakening of the plastic within weeks of exposure (as opposed to years) to outdoor conditions (Dijkstra et al., 2021). The rapid fragmentation also alleviates the aesthetically unacceptable macro-litter problem and the entanglement of marine animals by six-pack rings. The environmental impacts of making plastics photo-degrade faster depend on the trade-off between the increased ingestion risk posed by plastic fragments versus the potentially faster mineralization of the plastic debris (Dijkstra et al., 2021). The basic information needed to resolve this dilemma is, as yet, not available. Irrespectively, it can be argued that improved solid waste management practices, complemented by innovation in materials science that considers the full life cycle of the material used in a product, and which includes consideration of circularity, represents best practice to eliminate solid waste from entering the environment.

## 7. UV radiation environments relevant to plastics degradation

An important consideration when assessing the rate of degradation of plastics concerns their estimated exposure to UVR during use. For instance, PVC cladding material in buildings is routinely exposed to UVR, while underground plastic sewer pipes have very limited exposure. Plastic macrodebris dumped in landfills or buried in sediment, exclude any further exposure to UVR. It is the plastic litter on land and at sea that receives the highest amounts of UVR. For instance, smaller polymer fragments, including those from abrasive tire wear (Brahney et al., 2021), may become suspended in the atmosphere for extended periods of time (Allen et al., 2019; Allen et al., 2020; Evangeliou et al., 2020), where substantial exposure to UVR may occur. In aquatic environments, plastics that float or remain suspended at the water surface, are being transported towards shores, washed up on beaches, or released into the atmosphere by expulsion of bursting bubbles at the sea-air interface (Allen et al., 2020), all result in considerable exposure to UVR. However, the denser plastics, such as PS or PET, sink in water, precluding any UV-induced degradation. Based on exposure to UVR, the following zones for plastic materials can be identified:

1. Land-surfaces; UV irradiance is specific to location and time, and in general is relatively straightforward to estimate. The duration of exposure can also be estimated for each specific plastic application, ranging from fully exposed AstroTurf to buried water pipes.
2. Landfill sites; UVR does impinge on the surface of landfill. However, the penetration depth of UVR into the actual landfill mass is probably negligible given the soil cover on the fill will block most incoming radiation.
3. Water bodies. The penetration of UVR into waters depends to a large extent on the amounts of suspended particulates and other absorbers (e.g., DOM). Solar UVR penetration depths can vary considerably between different natural waters (Tedetti and Sempéré, 2006).
4. The atmosphere. This is typically the most intense UVR environment experienced by MPs. The amount of UVR present in the atmosphere is well understood both in theory and via measurements, as well as in spectral detail. Exposure duration is typically limited to a few weeks, depending on particle size.
5. Beaches and other secondary land sediments. As with landfills, the penetration of UVR is limited to the surface layers, with depths comparable to typical grain sizes, e.g. order of a few millimetres (Kühl et al., 1994). However, wave (and wind) action can create vertical mixing in the sand so that deeper MPs could find their way back to the surface after some time. The net duration of this exposure is unclear, although it may lend itself to certain measurements and modeling.

The five UVR environments are all disparate, and plastics will be exposed to different levels of UVR, different temperatures, different levels of access to atmospheric oxygen, and different levels of mechanical stress. Therefore, the persistence of plastics is a relative process, influenced by location-specific environmental conditions.

## 8. Degradation and fragmentation in a biological world

In comparison to photo-degradation, which is a well-documented (Fig. 2), both the underlying mechanism and the relative environmental importance of biodegradation of plastics remain to be established. Biodegradation typically comprises three distinct steps: 1) the formation of a biofilm on the surface of the plastic; 2) breakdown of the plastic into smaller molecules through the action of extracellular enzymes secreted by microorganisms; and 3) ingestion and further metabolism of these smaller molecules within the cell. Complex, multispecies biofilms develop on the surface of marine plastic debris (Kaiser et al., 2017) and the associated microbial community has been referred to as the “plastisphere” (Zettler et al., 2013). The “plastisphere” is thought to include putative plastic-degrading species (Roager and Sonnenschein, 2019; Yuan et al., 2020). Multiple species of bacteria and fungi that have been assumed to degrade plastics have been isolated from environmental samples (Yuan et al., 2020). In many cases, evidence for a particular biodegradation pathway is inferred based on laboratory studies, with limited data on the global relevance of identified processes. Plastics represent a heterogeneous mixture of varying polymer composition and associated additives. Consequently, biodegradation has been associated with a multitude of different reactions and enzyme systems (Krueger et al., 2015; Jacquin et al., 2019; Yuan et al., 2020). For example, degradation of PP, PE and PS can be mediated by mono-oxygenases, di-oxygenases, laccases, peroxidases and/or cutinases, which oxidize polymers to form more hydrophilic, low molecular weight compounds (Jacquin et al., 2019). Smaller, low molecular weight compounds such as plastic oligomers formed after hydrolysis, are amenable to further breakdown by an array of enzymes that can drive the formation of acetyl coA and succinyl coA. These compounds, in turn, can be metabolized via the tricarboxylic acid cycle completing the mineralization of plastics into CO_2_ and H_2_O within the cell (Jacquin et al., 2019). The environmentally relevant endpoint of such biodegradation is mineralization or the conversion of the polymer into small molecules such as CO_2_, CH_4_, water, or hydrogen. Some laboratory studies do indeed report aerobic mineralization of some classes of plastics. However, these studies are primarily on aerobic biodegradation in the epi-pelagic zone (light exposed), whereas most plastics end up in the anaerobic benthic sediment (Lott et al., 2021). Thus, a pertinent question concerns the relevance of biodegradation in the natural environment.

Although not a prerequisite for biodegradation, it is of interest to consider the role of photodegradation in facilitating the biological breakdown of plastic debris in the environment. There may be potential synergy between photo-degradation and biodegradation, due to the development of an oxygenated, hydrophilic surface layer on plastic debris due to photo-oxidation (Gewert et al., 2015; Yuan et al., 2020; Corti et al., 2010; Arkatkar et al., 2009; Ghatge et al., 2020). Furthermore, the formation of cracks and surface irregularities may encourage the establishment of a more abundant biofilm, by increasing the relative surface area of a particle. The formation of oligomers and or other small-molecular products (Dees et al., 2020) may also trigger further microbial biodegradation (Sivan et al., 2006). This phenomenon has been reported for UV irradiated PE exposed to specific microorganisms (Gilan et al., 2004; Hadad et al., 2005; Yamada-Onodera et al., 2001; Balasubramanian et al., 2014). However, even with photo-degradable PE mulch films used in agriculture, the observed combination of photo- and biodegradation does not proceed to mineralization under natural conditions (Vazquez et al., 2019). Even severe photo-oxidation, achieved by exposure of 20-micron PE film to germicidal UV-C resulted in just a two-fold stimulation of biodegradation by two strains of *Aspergillus* sp. and *Lysinibacillus* sp. Thus, it remains to be shown whether exposure to solar UVR in the natural environment is substantial enough to enhance biodegradation. In fact, the reverse may be more likely; photo-degradation may expedite biofilm formation, leading to faster sinking of debris to the marine bottom anaerobic sediment where degradation is likely minimal.

Purely mechanical fragmentation of ingested plastic material is an interesting source of MPs and NPs (Fig. 2). Dawson et al. (2018) first reported fragmentation of pristine plastics ingested by Antarctic krill. Since then, evidence concerning fragmentation of plastics by various organisms has rapidly expanded. The widespread, freshwater amphipod *Gammarus duebeni* fragments pristine polyethylene microplastics on a time scale of hours (Mateos-Cárdenas et al., 2020). In fact, more than 60 % of all detected microplastic particles in the amphipods’ digestive tracts were fragments, including particles in the nano-size range and which therefore can pass cell wall barriers and produce adverse effects on microalgae (Besseling et al., 2014), aquatic macrophytes (van Weert et al., 2019) or daphnids (Cui et al., 2017). Thus, biological fragmentation of plastics is potentially a codeterminant of the trophic mobility of plastics (Hasegawa and Nakaoka, 2021), although the quantitative scale of the phenomenon remains to be established.

## 9. Conclusions

UVR facilitated photo-oxidation drives the degradation of plastic debris, rendering it prone to subsequent fragmentation by mechanical stress. In the ocean, fragmentation is also driven by hydrodynamic forces, for example, in the beach ‘slush’ zone where waves break. While such forces can also fragment un-oxidized plastics, oxidative weathering makes common plastics weaker and more susceptible to fragmentation. While the qualitative features of this process are recognized, there are large gaps in knowledge, including the wavelength sensitivity and the dose-response functions for both oxidation and fragmentation processes. This lack of information coupled with the heterogeneity of the environment and the variability in plastic composition, which in turn affect rates of oxidative fragmentation, has precluded reliable quantitative models of plastic persistence being developed, especially at the global scale.

Given the primacy of solar UVR in weathering and fragmentation of plastics, the decrease in ground-level UV radiation consequent to the implementation of the Montreal Protocol plays a key role in determining plastic persistence. The “world avoided” refers to the success of the Montreal Protocol in avoiding high UV-B irradiances (McKenzie et al., 2019). The avoidance of the steep rises in UV radiation will have decreased the potential oxidation and fragmentation of plastic debris. In parallel, an emerging future perspective is that extensive mapping of global UV indices, undertaken in the context of the implementation of the Montreal Protocol, can be exploited to quantitatively model plastic weathering and fragmentation as a function of different geographic, climate and environmental zones, thereby informing management of plastic debris in the environment.

## Data Availability

No data was used for the research described in the article.

## HIGHLIGHTS

- Data on the fate of environmental plastic are needed to assess biological impacts.
- Photo-oxidation of plastic by solar UV radiation makes it prone to fragmentation.
- Implementation of the Montreal Protocol has consequences for plastic degradation.
- UV-driven plastic degradation varies between locations and environments.
- Knowledge gaps exist on wavelength sensitivity and dose-response of degradation.

## References

[R1] Alimi OS, Claveau-Mallet D, Kurusu RS, Lapointe M, Bayen S, Tufenkji N, 2022. Weathering pathways and protocols for environmentally relevant microplastics and nanoplastics: what are we missing? J. Hazard. Mater. 423, 126955. 34488100 10.1016/j.jhazmat.2021.126955

[R2] Allen S, Allen D, Phoenix VR, Le Roux G, Durántez Jiménez P, Simonneau A, Binet S, Galop D, 2019. Atmospheric transport and deposition of microplastics in a remote mountain catchment. Nat. Geosci. 12 (5), 339–344.

[R3] Allen S, Allen D, Moss K, Le Roux G, Phoenix VR, Sonke JE, 2020. Examination of the ocean as a source for atmospheric microplastics. PloS one 15 (5), e0232746. 32396561 10.1371/journal.pone.0232746 PMC7217454

[R4] Amaral-Zettler LA, Zettler ER, Slikas B, Boyd GD, Melvin DW, Morrall CE, Proskurowski G, Mincer TJ, 2015. The biogeography of the plastisphere: implications for policy. Front. Ecol. Environ. 13 (10), 541–546.

[R5] Andrady AL, 1997. Wavelength sensitivity in polymer photodegradation. Polym. Anal. Polym. Phys. 47–94.

[R6] Andrady AL, 2011. Microplastics in the marine environment. Mar. Pollut. Bull. 62 (8), 1596–1605. 21742351 10.1016/j.marpolbul.2011.05.030

[R7] Andrady AL, 2017. The plastic in microplastics: a review. Mar. Pollut. Bull. 119 (1), 12–22. 28449819 10.1016/j.marpolbul.2017.01.082

[R8] Andrady AL, 2022. Weathering and fragmentation of plastic debris in the ocean environment. Mar. Pollut. Bull. 180, 113761. 35665618 10.1016/j.marpolbul.2022.113761

[R9] Andrady AL, Law KL, Donohue J, Koongolla B, 2022. Accelerated degradation of low-density polyethylene in air and in sea water. Sci. Total Environ. 811, 151368. 34732340 10.1016/j.scitotenv.2021.151368

[R10] Anjana K, Hinduja M, Sujitha K, Dharani G, 2020. Review on plastic wastes in marine environment–biodegradation and biotechnological solutions. Mar. Pollut. Bull. 150, 110733. 31767203 10.1016/j.marpolbul.2019.110733

[R11] Arias-Villamizar CA, Vázquez-Morillas A, 2018. Degradation of conventional and oxodegradable high density polyethylene in tropical aqueous and outdoor environments. Rev. Int. Contam. Ambient. 34 (1), 137–147.

[R12] Arkatkar A, Arutchelvi J, Sudhakar M, Bhaduri S, Uppara PV, Doble M, 2009. Approaches to enhance the biodegradation of polyolefins. Open Environ. Eng. J. 2 (1).

[R13] Bais AF, Bernhard G, McKenzie RL, Aucamp PJ, Young PJ, Ilyas M, Jöckel P, Deushi M, 2019. Ozone–climate interactions and effects on solar ultraviolet radiation. Photochem. Photobiol. Sci. 18 (3), 602–640. 30810565 10.1039/c8pp90059k

[R14] Balasubramanian V, Natarajan K, Rajeshkannan V, Perumal P, 2014. Enhancement of in vitro high-density polyethylene (HDPE) degradation by physical, chemical, and biological treatments. Environ. Sci. Pollut. Res. 21 (21), 12549–12562. 10.1007/s11356-014-3191-2 24946709

[R15] Barnes PW, Robson TM, Neale PJ, Williamson CE, Zepp RG, Madronich S, Wilson SR, Andrady AL, Heikkilä AM, Bernhard GH, Bais AF, 2022. Environmental effects of stratospheric ozone depletion, UV radiation, and interactions with climate change: UNEP environmental effects assessment panel, update 2021. Photochem. Photobiol. Sci. 21 (3), 275–301. 35191005 10.1007/s43630-022-00176-5 PMC8860140

[R16] Besseling E, Wang B, Lürling M, Koelmans AA, 2014. Nanoplastic affects growth of S. obliquus and reproduction of D. magna. Environ. Sci. Technol. 48 (20), 12336–12343. 25268330 10.1021/es503001d PMC6863593

[R17] Besseling E, Quik JT, Sun M, Koelmans AA, 2017. Fate of nano-and microplastic in freshwater systems: a modeling study. Environ. Pollut. 220, 540–548. 27743792 10.1016/j.envpol.2016.10.001

[R18] Biber NF, Foggo A, Thompson RC, 2019. Characterising the deterioration of different plastics in air and seawater. Mar. Pollut. Bull. 141, 595–602. 30955772 10.1016/j.marpolbul.2019.02.068

[R19] Borrelle SB, Ringma J, Law KL, Monnahan CC, Lebreton L, McGivern A, Murphy E, Jambeck J, Leonard GH, Hilleary MA, Eriksen M, 2020. Predicted growth in plastic waste exceeds efforts to mitigate plastic pollution. Science 369 (6510), 1515–1518. 32943526 10.1126/science.aba3656

[R20] Bracco P, Costa L, Luda MP, Billingham N, 2018. A review of experimental studies of the role of free-radicals in polyethylene oxidation. Polym. Degrad. Stab. 155, 67–83.

[R21] Brahney J, Mahowald N, Prank M, Cornwell G, Klimont Z, Matsui H, Prather KA, 2021. Constraining the atmospheric limb of the plastic cycle. Proc. Natl. Acad. Sci. 118 (16). 10.1073/pnas.2020719118 PMC8072239 33846251

[R22] Cai L, Wang J, Peng J, Wu Z, Tan X, 2018. Observation of the degradation of three types of plastic pellets exposed to UV irradiation in three different environments. Sci. Total Environ. 628, 740–747. 29454214 10.1016/j.scitotenv.2018.02.079

[R23] Chubarenko I, Efimova I, Bagaeva M, Bagaev A, Isachenko I, 2020. On mechanical fragmentation of single-use plastics in the sea swash zone with different types of bottom sediments: insights from laboratory experiments. Mar. Pollut. Bull. 150, 110726. 31780093 10.1016/j.marpolbul.2019.110726

[R24] Coffin S, Wyer H, Leapman JC, 2021. Addressing the environmental and health impacts of microplastics requires open collaboration between diverse sectors. PLoS Biol. 19 (3), e3000932. 33784313 10.1371/journal.pbio.3000932 PMC8009430

[R25] Corcoran PL, Biesinger MC, Grifi M, 2009. Plastics and beaches: a degrading relationship. Mar. Pollut. Bull. 58 (1), 80–84. 18834997 10.1016/j.marpolbul.2008.08.022

[R26] Corti A, Muniyasamy S, Vitali M, Imam SH, Chiellini E, 2010. Oxidation and biodegradation of polyethylene films containing pro-oxidant additives: synergistic effects of sunlight exposure, thermal aging and fungal biodegradation. Polym. Degrad. Stab. 95 (6), 1106–1114.

[R27] Cui R, Kim SW, An YJ, 2017. Polystyrene nanoplastics inhibit reproduction and induce abnormal embryonic development in the freshwater crustacean Daphnia galeata. Sci. Rep. 7 (1), 1–10. 28935955 10.1038/s41598-017-12299-2 PMC5608696

[R28] Dawson AL, Kawaguchi S, King CK, Townsend KA, King R, Huston WM, Bengtson Nash SM, 2018. Turning microplastics into nanoplastics through digestive fragmentation by Antarctic krill. Nat. Commun. 9 (1), 1–8. 29520086 10.1038/s41467-018-03465-9 PMC5843626

[R29] de Carvalho CC, 2017. Biofilms: microbial strategies for surviving UV exposure. Ultraviolet Light in Human Health, Diseases and Environment, pp. 233–239. 10.1007/978-3-319-56017-5_19 29124704

[R30] De Falco F, Cocca M, Avella M, Thompson RC, 2020. Microfiber release to water, via laundering, and to air, via everyday use: a comparison between polyester clothing with differing textile parameters. Environ. Sci. Technol. 54 (6), 3288–3296. 32101431 10.1021/acs.est.9b06892

[R31] Dees JP, Ateia M, Sanchez DL, 2020. Microplastics and their degradation products in surface waters: a missing piece of the global carbon cycle puzzle. ACS ES&T Water 1 (2), 214–216.

[R32] Dijkstra H, van Beukering P, Brouwer R, 2021. In the business of dirty oceans: overview of startups and entrepreneurs managing marine plastic. Mar. Pollut. Bull. 162, 111880. 33307401 10.1016/j.marpolbul.2020.111880

[R33] Evangeliou N, Grythe H, Klimont Z, Heyes C, Eckhardt S, Lopez-Aparicio S, Stohl A, 2020. Atmospheric transport is a major pathway of microplastics to remote regions. Nat. Commun. 11 (1), 1–11. 32665541 10.1038/s41467-020-17201-9 PMC7360784

[R34] Fauser P, Vorkamp K, Strand J, 2022. Residual additives in marine microplastics and their risk assessment–a critical review. Mar. Pollut. Bull. 177, 113467. 35314391 10.1016/j.marpolbul.2022.113467

[R35] Gago J, Carretero O, Filgueiras AV, Viñas L, 2018. Synthetic microfibers in the marine environment: a review on their occurrence in seawater and sediments. Mar. Pollut. Bull. 127, 365–376. 29475673 10.1016/j.marpolbul.2017.11.070

[R36] Garton A, Carlsson DJ, Wiles DM, 1980. Polymer oxidation and secondary cage combination of peroxyl radicals. Makromol. Chem. Macromol. Chem. Phys. 181 (9), 1841–1846.

[R37] Garvey CJ, Impéror-Clerc M, Rouzière S, Gouadec G, Boyron O, Rowenczyk L, Mingotaud AF, Ter Halle A, 2020. Molecular-scale understanding of the embrittlement in polyethylene ocean debris. Environ. Sci. Technol. 54 (18), 11173–11181. 32808772 10.1021/acs.est.0c02095

[R38] Gewert B, Plassmann MM, MacLeod M, 2015. Pathways for degradation of plastic polymers floating in the marine environment. Environ Sci Process Impacts 17 (9), 1513–1521. 26216708 10.1039/c5em00207a

[R39] Ghatge S, Yang Y, Ahn JH, Hur HG, 2020. Biodegradation of polyethylene: a brief review. Appl. Biol. Chem. 63 (1), 1–14.

[R40] Gilan I, Hadar Y, Sivan A, 2004. Colonization, biofilm formation and biodegradation of polyethylene by a strain of rhodococcus ruber. Appl. Microbiol. Biotechnol. 65 (1), 97–104. 15221232 10.1007/s00253-004-1584-8

[R41] Girois S, Audouin L, Verdu J, Delprat P, Marot G, 1996. Molecular weight changes during the photooxidation of isotactic polypropylene. Polym. Degrad. Stab. 51 (2), 125–132.

[R42] Gouin T, 2021. Addressing the importance of microplastic particles as vectors for long-range transport of chemical contaminants: perspective in relation to prioritizing research and regulatory actions. Microplast. Nanoplast. 1 (1), 1–19.

[R43] Grause G, Chien MF, Inoue C, 2020. Changes during the weathering of polyolefins. Polym. Degrad. Stab. 181, 109364.

[R44] Hadad D, Geresh S, Sivan A, 2005. Biodegradation of polyethylene by the thermophilic bacterium brevibacillus borstelensis. J. Appl. Microbiol. 98 (5), 1093–1100. 15836478 10.1111/j.1365-2672.2005.02553.x

[R45] Hakkarainen M, Albertsson AC, 2004. Environmental degradation of polyethylene. Long Term Properties of Polyolefins, pp. 177–200.

[R46] Harris PT, 2020. The fate of microplastic in marine sedimentary environments: a review and synthesis. Mar. Pollut. Bull. 158, 111398. 32753183 10.1016/j.marpolbul.2020.111398

[R47] Hasegawa T, Nakaoka M, 2021. Trophic transfer of microplastics from mysids to fish greatly exceeds direct ingestion from the water column. Environ. Pollut. 273, 116468. 33477061 10.1016/j.envpol.2021.116468

[R48] Hodgson DJ, Bréchon AL, Thompson RC, 2018. Ingestion and fragmentation of plastic carrier bags by the amphipod orchestia gammarellus: effects of plastic type and fouling load. Mar. Pollut. Bull. 127, 154–159. 29475648 10.1016/j.marpolbul.2017.11.057

[R49] Ito M, Masuda Y, Nagai K, 2015. Evaluation of long-term stability and degradation on polycarbonate based plastic glass. J. Polym. Eng. 35 (1), 31–40.

[R50] Jacquin J, Cheng J, Odobel C, Pandin C, Conan P, Pujo-Pay M, Barbe V, Meistertzheim AL, Ghiglione JF, 2019. Microbial ecotoxicology of marine plastic debris: a review on colonization and biodegradation by the “Plastisphere”. Front. Microbiol. 10, 865. 31073297 10.3389/fmicb.2019.00865 PMC6497127

[R51] Kaiser D, Kowalski N, Waniek JJ, 2017. Effects of biofouling on the sinking behavior of microplastics. Environ. Res. Lett. 12 (12), 124003.

[R52] Kalogerakis N, Karkanorachaki K, Kalogerakis GC, Triantafyllidi EI, Gotsis AD, Partsinevelos P, Fava F, 2017. Microplastics generation: onset of fragmentation of polyethylene films in marine environment mesocosms. Front. Mar. Sci. 4, 84.

[R53] Karlsson TM, Hassellöv M, Jakubowicz I, 2018. Influence of thermooxidative degradation on the in situ fate of polyethylene in temperate coastal waters. Mar. Pollut. Bull. 135, 187–194. 30301030 10.1016/j.marpolbul.2018.07.015

[R54] Koelmans B, Pahl S, Backhaus T, Bessa F, van Calster G, Contzen N, Cronin R, Galloway T, Hart A, Henderson L, Kalcikova G, 2019. A Scientific Perspective on Microplastics in Nature and Society. SAPEA.

[R55] Krueger MC, Harms H, Schlosser D, 2015. Prospects for microbiological solutions to environmental pollution with plastics. Appl. Microbiol. Biotechnol. 99 (21), 8857–8874. 26318446 10.1007/s00253-015-6879-4

[R56] Kühl M, Lassen C, Jørgensen BB, 1994. Light penetration and light intensity in sandy marine sediments measured with irradiance and scalar irradiance fiber-optic microprobes. Mar. Ecol. Prog. Ser. 139–148.

[R57] Lambert S, Wagner M, 2016a. Formation of microscopic particles during the degradation of different polymers. Chemosphere 161, 510–517. 27470943 10.1016/j.chemosphere.2016.07.042

[R58] Lambert S, Wagner M, 2016b. Characterisation of nanoplastics during the degradation of polystyrene. Chemosphere 145, 265–268. 26688263 10.1016/j.chemosphere.2015.11.078 PMC5250697

[R59] Lebreton L, Andrady A, 2019. Future scenarios of global plastic waste generation and disposal. Palgrave Commun. 5 (1), 1–11.

[R60] Lebreton L, Egger M, Slat B, 2019. A global mass budget for positively buoyant macroplastic debris in the ocean. Sci. Rep. 9 (1), 1–10. 31515537 10.1038/s41598-019-49413-5 PMC6742645

[R61] Leiser R, Jongsma R, Bakenhus I, Möckel R, Philipp B, Neu TR, Wendt-Potthoff K, 2021. Interaction of cyanobacteria with calcium facilitates the sedimentation of microplastics in a eutrophic reservoir. Water Res. 189, 116582. 33166918 10.1016/j.watres.2020.116582

[R62] Lott C, Eich A, Makarow D, Unger B, Van Eekert M, Schuman E, Reinach MS, Lasut MT, Weber M, 2021. Half-life of biodegradable plastics in the marine environment depends on material, habitat, and climate zone. Front. Mar. Sci. 8, 426.

[R63] Masry M, Rossignol S, Gardette JL, Therias S, Bussière PO, Wong-Wah-Chung P, 2021. Characteristics, fate, and impact of marine plastic debris exposed to sunlight: a review. Mar. Pollut. Bull. 171, 112701. 34245992 10.1016/j.marpolbul.2021.112701

[R64] Mateos-Cárdenas A, O’Halloran J, van Pelt FN, Jansen MA, 2020. Rapid fragmentation of microplastics by the freshwater amphipod Gammarus duebeni (Lillj.). Sci. Rep. 10 (1), 1–12. 32732882 10.1038/s41598-020-69635-2 PMC7393071

[R65] McKenzie R, Bernhard G, Liley B, Disterhoft P, Rhodes S, Bais A, Morgenstern O, Newman P, Oman L, Brogniez C, Simic S, 2019. Success of Montreal protocol demonstrated by comparing high-quality UV measurements with “World avoided” calculations from two chemistry-climate models. Sci. Rep. 9 (1), 1–13. 31481668 10.1038/s41598-019-48625-z PMC6722083

[R66] Mehinto AC, Coffin S, Koelmans AA, Brander SM, Wagner M, Thornton Hampton LM, Burton AG, Miller E, Gouin T, Weisberg SB, Rochman CM, 2022. Risk-based management framework for microplastics in aquatic ecosystems. Microplast. Nanoplast. 2 (1), 1–10.35005629

[R67] Nabi I, Ahmad F, Zhang L, 2021. Application of titanium dioxide for the photocatalytic degradation of macro-and micro-plastics: a review. J. Environ. Chem. Eng. 9 (5), 105964.

[R68] Nagai N, Matsunobe T, Imai T, 2005. Infrared analysis of depth profiles in UV-photochemical degradation of polymers. Polym. Degrad. Stab. 88 (2), 224–233.

[R69] Neale RE, Barnes PW, Robson TM, Neale PJ, Williamson CE, Zepp RG, Wilson SR, Madronich S, Andrady AL, Heikkilä AM, Bernhard GH, 2021. Environmental effects of stratospheric ozone depletion, UV radiation, and interactions with climate change: UNEP environmental effects assessment panel, update 2020. Photochem. Photobiol. Sci. 20 (1), 1–67. 33721243 10.1007/s43630-020-00001-x PMC7816068

[R70] Ojeda T, Freitas A, Birck K, Dalmolin E, Jacques R, Bento F, Camargo F, 2011. Degradability of linear polyolefins under natural weathering. Polym. Degrad. Stab. 96 (4), 703–707.

[R71] Onink V, Jongedijk CE, Hoffman MJ, van Sebille E, Laufkötter C, 2021. Global simulations of marine plastic transport show plastic trapping in coastal zones. Environ. Res. Lett. 16 (6), 064053.

[R72] Ouyang Z, Yang Y, Zhang C, Zhu S, Qin L, Wang W, He D, Zhou Y, Luo H, Qin F, 2021. Recent advances in photocatalytic degradation of plastics and plastic-derived chemicals. J. Mater. Chem. A 9 (23), 13402–13441.

[R73] Pabortsava K, Lampitt RS, 2020. High concentrations of plastic hidden beneath the surface of the Atlantic Ocean. Nat. Commun. 11 (1), 1–11. 32811835 10.1038/s41467-020-17932-9 PMC7434887

[R74] Petukhova ES, Fedorov AL, 2019. Investigation of the climate resistance of stabilized polyethylene composite materials. Procedia Struct. Integ. 20, 75–80.

[R75] Pickett JE, 2018. Weathering of plastics. Handbook of Environmental Degradation of Materials. William Andrew Publishing, pp. 163–184.

[R76] Póvoa AA, Skinner LF, de Araújo FV, 2021. Fouling organisms in marine litter (rafting on abiogenic substrates): a global review of literature. Mar. Pollut. Bull. 166, 112189. 33662701 10.1016/j.marpolbul.2021.112189

[R77] Rani M, Shim WJ, Han GM, Jang M, Song YK, Hong SH, 2017. Benzotriazole-type ultraviolet stabilizers and antioxidants in plastic marine debris and their new products. Sci. Total Environ. 579, 745–754. 27889215 10.1016/j.scitotenv.2016.11.033

[R78] Roager L, Sonnenschein EC, 2019. Bacterial candidates for colonization and degradation of marine plastic debris. Environ. Sci. Technol. 53 (20), 11636–11643. 31557003 10.1021/acs.est.9b02212

[R79] Rodriguez AK, Mansoor B, Ayoub G, Colin X, Benzerga AA, 2020. Effect of UV-aging on the mechanical and fracture behavior of low density polyethylene. Polym. Degrad. Stab. 180, 109185.

[R80] Schernewski G, Radtke H, Robbe E, Haseler M, Hauk R, Meyer L, Piehl S, Riedel J, Labrenz M, 2021. Emission, transport, and deposition of visible plastics in an estuary and the Baltic Sea—a monitoring and modeling approach. Environ. Manag. 68 (6), 860–881. 10.1007/s00267-021-01534-2 PMC8578054 34505927

[R81] Sendra M, Pereiro P, Figueras A, Novoa B, 2021. An integrative toxicogenomic analysis of plastic additives. J. Hazard. Mater. 409, 124975. 33388451 10.1016/j.jhazmat.2020.124975

[R82] Shah AA, Hasan F, Hameed A, Ahmed S, 2008. Biological degradation of plastics: a comprehensive review. Biotechnol. Adv. 26 (3), 246–265. 18337047 10.1016/j.biotechadv.2007.12.005

[R83] Shyichuk AV, Turton TJ, White JR, Syrotynska ID, 2004. Different degradability of two similar polypropylenes as revealed by macromolecule scission and crosslinking rates. Polym. Degrad. Stab. 86 (2), 377–383.

[R84] Sivan A, Szanto M, Pavlov V, 2006. Biofilm development of the polyethylene-degrading bacterium rhodococcus ruber. Appl. Microbiol. Biotechnol. 72 (2), 346–352. 16534612 10.1007/s00253-005-0259-4

[R85] Song YK, Hong SH, Jang M, Han GM, Jung SW, Shim WJ, 2017. Combined effects of UV exposure duration and mechanical abrasion on microplastic fragmentation by polymer type. Environ. Sci. Technol. 51 (8), 4368–4376. 28249388 10.1021/acs.est.6b06155

[R86] Stubbins A, Law KL, Muñoz SE, Bianchi TS, Zhu L, 2021. Plastics in the earth system. Science 373 (6550), 51–55. 34210876 10.1126/science.abb0354

[R87] Suaria G, Achtypi A, Perold V, Lee JR, Pierucci A, Bornman TG, Aliani S, Ryan PG, 2020. Microfibers in oceanic surface waters: a global characterization. Sci. Adv. 6 (23), 8493. 10.1126/sciadv.aay8493 PMC7274779 32548254

[R88] Sun J, Zhu ZR, Li WH, Yan X, Wang LK, Zhang L, Jin J, Dai X, Ni BJ, 2021. Revisiting microplastics in landfill leachate: unnoticed tiny microplastics and their fate in treatment works. Water Res. 190, 116784. 33387953 10.1016/j.watres.2020.116784

[R89] Svedin J, 2020. Photodegradation of Macroplastics to Microplastics: A Laboratory Study on Common Litter Found in Urban Areas. University of Technology, Sweden M. Thesis Department of Natural Resource Engineering, Lule.

[R90] Tamblyn JW, Newland GC, 1965. Induction period in the aging of polypropylene. J. Appl. Polym. Sci. 9 (6), 2251–2260.

[R91] Tedetti M, Sempéré R, 2006. Penetration of ultraviolet radiation in the marine environment. A review. Photochem. Photobiol. 82 (2), 389–397. 16613490 10.1562/2005-11-09-IR-733

[R92] Ter Halle A, Ladirat L, Martignac M, Mingotaud AF, Boyron O, Perez E, 2017. To what extent are microplastics from the open ocean weathered? Environ. Pollut. 227, 167–174. 28460234 10.1016/j.envpol.2017.04.051

[R93] Therias S, Rapp G, Masson C, Gardette JL, 2021. Limits of UV-light acceleration on the photooxidation of low-density polyethylene. Polym. Degrad. Stab. 183, 109443.

[R94] Tramoy R, Gasperi J, Colasse L, Tassin B, 2020. Transfer dynamic of macroplastics in estuaries—new insights from the seine estuary: part 1. Long term dynamic based on date-prints on stranded debris. Mar. Pollut. Bull. 152, 110894. 31957678 10.1016/j.marpolbul.2020.110894

[R95] Van Melkebeke M, Janssen C, De Meester S, 2020. Characteristics and sinking behavior of typical microplastics including the potential effect of biofouling: implications for remediation. Environ. Sci. Technol. 54 (14), 8668–8680. 32551546 10.1021/acs.est.9b07378

[R96] van Weert S, Redondo-Hasselerharm PE, Diepens NJ, Koelmans AA, 2019. Effects of nanoplastics and microplastics on the growth of sediment-rooted macrophytes. Sci. Total Environ. 654, 1040–1047. 30841378 10.1016/j.scitotenv.2018.11.183

[R97] Vassilenko E, Watkins M, Chastain S, Mertens J, Posacka AM, Patankar S, Ross PS, 2021. Domestic laundry and microfiber pollution: exploring fiber shedding from consumer apparel textiles. Plos one 16 (7), e0250346. 34242234 10.1371/journal.pone.0250346 PMC8270180

[R98] Vazquez YV, Ressia JA, Cerrada ML, Barbosa SE, Valles EM, 2019. Prodegradant additives effect onto comercial polyolefins. J. Polym. Environ. 27 (3), 464–471.

[R99] Vega GC, Gross A, Birkved M, 2021. The impacts of plastic products on air pollution-a simulation study for advanced life cycle inventories of plastics covering secondary microplastic production. Sustain. Prod. Consum. 28, 848–865.

[R100] Ward CP, Reddy CM, 2020. Opinion: we need better data about the environmental persistence of plastic goods. Proc. Natl. Acad. Sci. 117 (26), 14618–14621. 32522872 10.1073/pnas.2008009117 PMC7334500

[R101] Ward CP, Armstrong CJ, Walsh AN, Jackson JH, Reddy CM, 2019. Sunlight converts polystyrene to carbon dioxide and dissolved organic carbon. Environ. Sci. Technol. Lett. 6 (11), 669–674.

[R102] Weiss L, Ludwig W, Heussner S, Canals M, Ghiglione JF, Estournel C, Constant M, Kerhervé P, 2021. The missing ocean plastic sink: gone with the rivers. Science 373 (6550), 107–111. 34210886 10.1126/science.abe0290

[R103] Woodall LC, Sanchez-Vidal A, Canals M, Paterson GL, Coppock R, Sleight V, Calafat A, Rogers AD, Narayanaswamy BE, Thompson RC, 2014. The deep sea is a major sink for microplastic debris. R. Soc. Open Sci. 1 (4), 140317. 26064573 10.1098/rsos.140317 PMC4448771

[R104] Wu P, Tang Y, Cao G, Li J, Wang S, Chang X, Dang M, Jin H, Zheng C, Cai Z, 2020. Determination of environmental micro (nano) plastics by matrix-assisted laser desorption/ionization–time-of-flight mass spectrometry. Anal. Chem. 92 (21), 14346–14356. 32880171 10.1021/acs.analchem.0c01928

[R105] Wypych G, 2020. Handbook of UV Degradation and Stabilization. Elsevier, Third ed. ChemTec Publishing, Toronto, p. 518.

[R106] Yamada-Onodera K, Mukumoto H, Katsuyaya Y, Saiganji A, Tani Y, 2001. Degradation of polyethylene by a fungus, penicillium simplicissimum YK. Polym. Degrad. Stab. 72 (2), 323–327.

[R107] Yuan J, Ma J, Sun Y, Zhou T, Zhao Y, Yu F, 2020. Microbial degradation and other environmental aspects of microplastics/plastics. Sci. Total Environ. 715, 136968. 32014782 10.1016/j.scitotenv.2020.136968

[R108] Zettler ER, Mincer TJ, Amaral-Zettler LA, 2013. Life in the “plastisphere”: microbial communities on plastic marine debris. Environ. Sci. Technol. 47 (13), 7137–7146. 23745679 10.1021/es401288x

[R109] Zhu X, 2021. The plastic cycle–an unknown branch of the carbon cycle. Front. Mar. Sci. 7, 1227.

[R110] Zhu L, Zhao S, Bittar TB, Stubbins A, Li D, 2020. Photochemical dissolution of buoyant microplastics to dissolved organic carbon: rates and microbial impacts. J. Hazard. Mater. 383, 121065. 31518809 10.1016/j.jhazmat.2019.121065

